# Collisional Quantum Dynamics for MgH^−^ (^1^Σ^+^) With He as a Buffer Gas: Ionic State-Changing Reactions in Cold Traps

**DOI:** 10.3389/fchem.2019.00064

**Published:** 2019-02-12

**Authors:** Lola González-Sánchez, Susana Gómez-Carrasco, Alberto M. Santadaría, Roland Wester, Francesco A. Gianturco

**Affiliations:** ^1^Departamento de Química Física, University of Salamanca, Salamanca, Spain; ^2^Institute of Chemistry, ELTE Eötvös Loránd University, Budapest, Hungary; ^3^Department of Physics, Institut für Ionenphysik und Angewandte Physik, Universitaet Innsbruck, Innsbruck, Austria

**Keywords:** molecular collisions, atom molecule interactions, rotational state changing dynamics, collisional cooling/heating in ion traps, kinetic states evolution

## Abstract

We present in this paper a detailed theoretical and computational analysis of the quantum inelastic dynamics involving the lower rotational levels of the MgH^−^ (X^1^Σ^+^) molecular anion in collision with He atoms which provide the buffer gas in a cold trap. The interaction potential between the molecular partner and the He (^1^*S*) gaseous atoms is obtained from accurate quantum chemical calculations at the post-Hartree-Fock level as described in this paper. The spatial features and the interaction strength of the present potential energy surface (PES) are analyzed in detail and in comparison with similar, earlier results involving the MgH^+^ (^1^Σ) cation interacting with He atoms. The quantum, multichannel dynamics is then carried out using the newly obtained PES and the final inelastic rats constants, over the range of temperatures which are expected to be present in a cold ion trap experiment, are obtained to generate the multichannel kinetics of population changes observed for the molecular ion during the collisional cooling process. The rotational populations finally achieved at specific temperatures are linked to state-selective laser photo-detachment experiments to be carried out in our laboratory.All intermediate steps of the quantum modeling are also compared with the behavior of the corresponding MgH^+^ cation in the trap and the marked differences which exist between the collisional dynamics of the two systems are dicussed and explained. The feasibility of the present anion to be involved in state-selective photo-detachment experiments is fully analyzed and suggestions are made for the best performing conditions to be selected during trap experiments.

## 1. Introduction

The investigation of chemical processes at very low temperature, and the analyses of the physics involved to understand their mechanism at the molecular level, has gone through a marked development in the last decade or so. The new scientific vistas offered by their findings and the many possibilities for various applications which have become associated with cold and ultracold molecules have been reported in detail in several recent reviews (Bell and Softley, 2009; Carr et al., 2009; Dulieu and Gabbanini, 2009; Gianturco and Tacconi, 2009; Herschbach, 2009; Krems et al., 2009; Bai et al., 2011; Quéméner and Julienne, 2012; Willitsch, 2017). We shall therefore not repeat here all the current motivations and all the types of opportunities discussed there, but simply note that such wealth of new information points at very pursuing effects in the domain of precision measurements, high-resolution spectroscopy for a surprising variety of molecular systems, quantum simulation and quantum computing by interrogation of cold, trapped molecular ions.

As an example, several experimental groups have combined the use of radio-frequency ion traps with the additional presence of optical dipole traps and also magneto-optical traps (Grier et al., 2009; Schmid et al., 2010; Hall et al., 2011; Rellergert et al., 2011; Hall and Willitsch, 2012; Ratschbacher et al., 2012) in order to obtain confinement of both cold molecular ions and cold atoms. Such successful achievements point at the real possibility of studying ionic chemical reactions in a new temperature domain down to a few millikelvin. Furthermore, the combination of crossed-beam experiments with three-dimensional velocity map imaging has allowed to elucidate, albeit at higher collision energies than that of the millikelvin, the dependence of charge-transfer processes on angular distributions and on the chosen vibrational state of the molecular partner in the reaction (Williams et al., 2018).

With the same token, the confinement down to temperatures of a few K of simple molecular anions in cold ion traps with the presence of He as a buffer gas has been another achievement for unraveling quantum effects, at the low temperature regimes, for specific molecular processes involving laser-assisted selective photo-detachment (Hauser et al., 2015; Hernández Vera et al., 2018; Lakhmanskaya et al., 2018). In that work, in fact, both the experiments and the calculations of OH^−^ and ${\text{NH}}_{2}^{-}$ anionic systems (trapped down to 10–20 K temperature regimes) have shown clearly that it is possible for such trapped molecules to be kept in specific rotational states of their ground electronic state and |*v*> = 0 vibrational state. Such selected initial conditions can then be used to study photo-detachment processes where only one or two rotational states are depleted from the trap by laser detachment of their access electron (Gianturco et al., 2018).

If therefore becomes of direct interest from the above findings to further investigate polar molecular anions which could be employed for direct selective photo-detachment experiments and also modeled from quantum simulations of their structural and dynamical aspects. We have recently studied in detail the electronic properties of the MgH^−^ (X^2^Σ^+^) anion and of its corresponding neutral (González-Sánchez et al., 2017), MgH (X^2^Σ^+^) with the aim of accurately assessing the adiabatic electron affinity (AEA) value of the anion, as well as which electronic states of both systems would become relevant during a photo-detachment experiment.

Since such experiments involve the preparation of the partner molecular anion in specific rotovibrational states of its ground electronic state (Lakhmanskaya et al., 2018), one further needs to realistically assess the corresponding efficiency of collisionally “cooling” the internal states of the molecular anion in a trap that employs He as a buffer gas. Hence, the present work intends to present in detail the interaction forces acting between an MgH^−^ molecular target in its |*v* >= 0 state and the He gas in the buffer role, populating by collisions a few of the lower rotational states of the anion within the trap.

We have recently carried out, in fact, a detailed calculation comparing the electronic properties and structural features of the isolated MgH^−^ anion and of its neutral counterpart, MgH (X^2^Σ^+^), as the two main partners of a selective photo-detachment experiment (González-Sánchez et al., 2017). In order to further investigate the experimental preparation of MgH^−^ molecules in specific rotational states, we now need to examine the dynamics of state-changing kinetics of MgH^−^ in a cold ion trap and under the presence of He gas as a buffer gas uploaded in the cold trap (Hauser et al., 2015; Hernández Vera et al., 2018).

To achieve such end, we therefore discuss in the following section 2 the structural features of the potential energy surface (PES) involving the MgH^−^ anionic molecule and the He atom. In particular, we shall describe the spatial anisotropy of such interaction and its effect on driving specific rotational state-changing inelastic collisions in the trap. The next section 3 will briefly outline our quantum treatment of the time-independent description of the scattering events within the multichannel formulation of the Schrödinger Equation (TISE) and present our results for a broad range of inelastic cross sections, computed at the relative collision energies which map those present in the cold trap regime. We shall further analyse those data in order to extract specific propensity rules on the relative sizes of the transition probabilities during collisions.

The ensuing section 4 will report the computed state-to-state inelastic rates over the relevant range of temperatures. Our final considerations and conclusions will be given by the last section 5.

## 2. Interaction Potential and Spatial Anisotropy of the PES

All calculations have been performed using a *C*_*s*_ point group of symmetry. The aug-cc-pV5Z basis set has been used for both H and He. The core-valence aug-cc-pwCV5Z basis set has been used for the Mg atom. A standard Hartree Fock calculation is initially done, followed next by a complete active space calculation (CASSCF) in which 6 active electrons are distributed among 11 active orbitals (8a' and 3a”). The latter correspond to the 3s, 3p, and 3d orbitals for the Mg atom plus the 1s orbitals for H and He. In all calculations, the core orbitals (4a' and 1a”) are kept doubly occupied. After that, a multi-reference configuration interaction (MRCI) calculation is done including single and double excitations and a perturbative estimation for higher order excitation (Davidson correction).

The calculations are done in bond coordinates, Mg-H, H-He, and the angle Mg-H-He.The grid includes 13 angular points, 20 radial points for the Mg-H distance and 37 radial points for the H-He distance.

The above points where in turn transformed into (*r, R*, θ) Jacobi coordinates in order to carry out the quantum scattering calculations (see below). As a first instance, we have treated the dynamics using a Rigid-Rotor (RR) description of the target molecular ion. Hence, the RR grid involved the (*r*_*eq*_, *R*, θ) set of points, for which we generated 37 points in *R* and 15 points for θ. The *r*_*eq*_=1.7368 Å as discussed in González-Sánchez et al. (2017) and all calculations were carried out using the MOLPRO computational package Werner et al. (2015).

**Figure 1:**
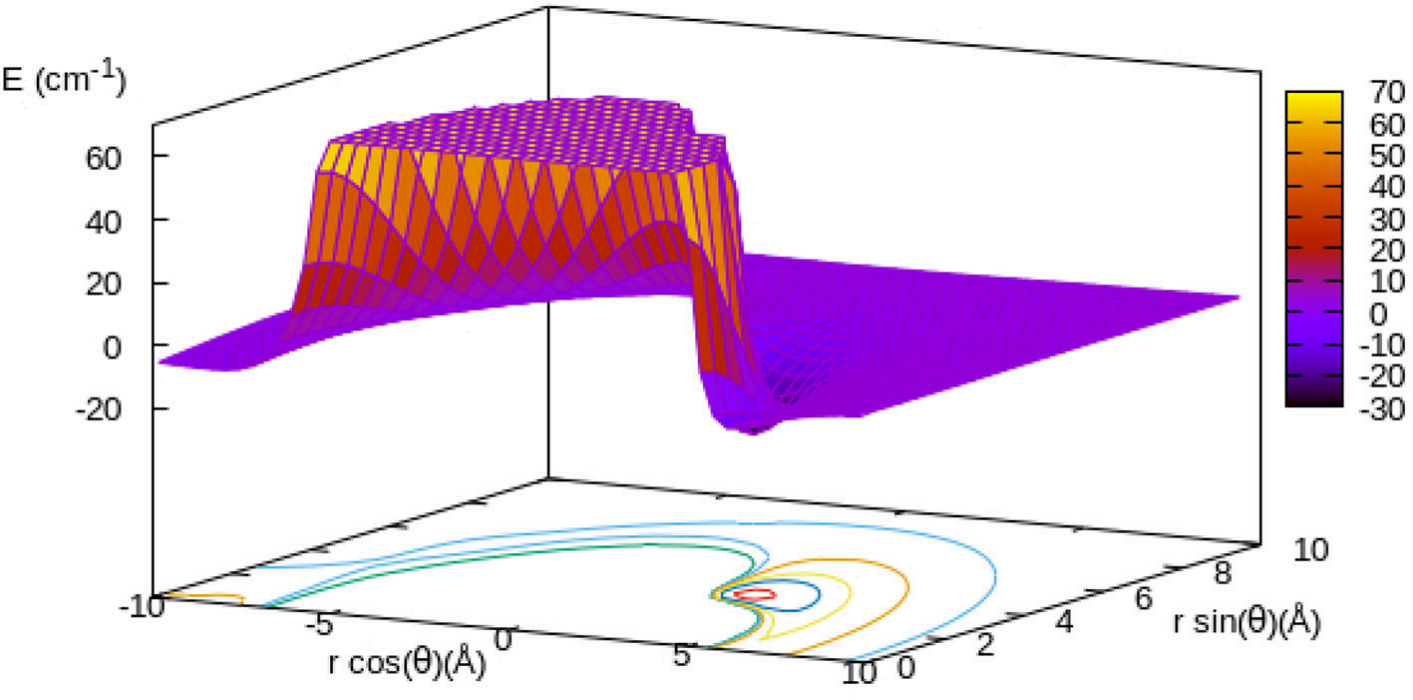
Computed PES for the *r*_*eq*_ geometry of MgH^−^ (see main text) interacting with He. Distances in Å, energy in cm^−1^. The Mg atom on the negative axis.

The data shown by Figure 1 report the spatial features of the potential energy surface (PES) that describes the RR MgH^−^ anion interacting with the He atom.

The Mg^−^ end of the molecule is placed on the negative region of the projection plane, while the H atom is next to the off-axes location of the attractive well region of the PES. The excess electron is clearly largely located on the Mg-side of the molecular ion and therefore the strongest interaction with the He atom is, as expected, around the H-region of the molecular anion.

Since we have previously analyzed a similar PES involving the MgH^+^ cation in its X^1^Σ^+^ electronic state (Tacconi et al., 2011; Caruso et al., 2012), it is interesting to compare the two types of interaction potentials to extract further structural information on the present system.

**Figure 2:**
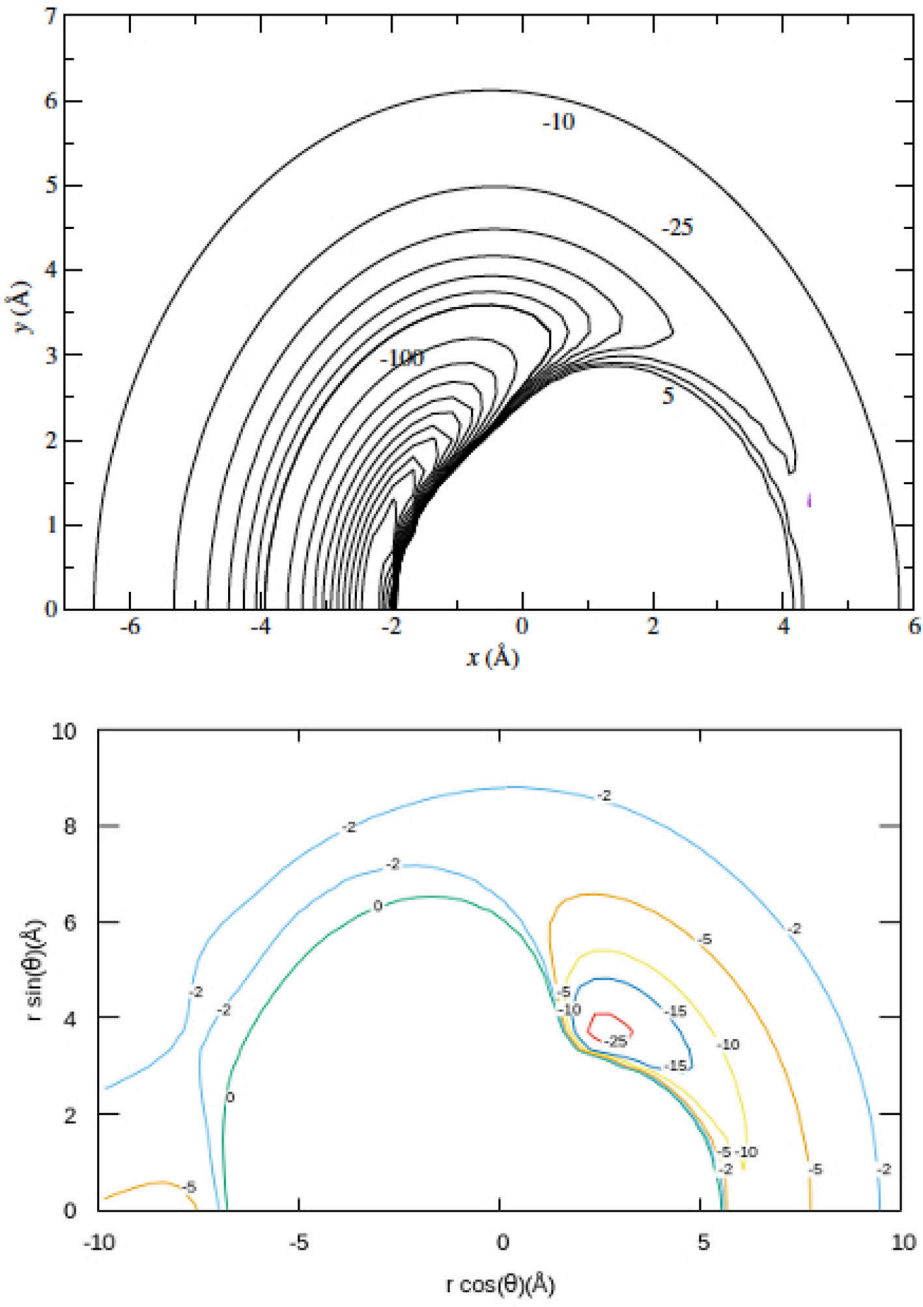
In-plane projections of the PESs associated with the MgH^+^/He **(Upper)** and MgH^−^/He **(Lower)**. See main text for details.

The panels shown by Figure 2 report the on-plane projections of the PES associated to the MgH^+^ (X^1^Σ^+^) with He (upper panel) and the one describing the MgH^−^ (X^1^Σ^+^) with He in the lower panels. Both Mg-ends of the molecules are on the negative region of the x/*r*cos(θ) axis in the panels. Distances in Å and energy levels in cm^−1^.

One clearly see by comparison that the cationic partner present a stronger interaction with the He atom: the latter is markedly attracted on the Mg-end of the molecular cation while it shows its weaker attractive well for the anion on the H-end of the MgH^−^ molecule. Furthermore, we see that the present anion exhibits a large region round the molecule where the interaction remains repulsive, while He gets much closer to the Mg region in the case of the cation, as shown the upper panel. Such differences are clearly linked with the extra negative charge bound to the MgH^−^ partner with respect to the cation, as we shall further analyse below.

As we shall discuss in the next section, the spatial anisotropy of the interaction, i.e., its strength over radial range and its dependence on the orientational Jacobi angle θ, are important markers for the dynamical torque which is being applied to the rotating molecule as it collides with the He atom (Tacconi et al., 2011; Caruso et al., 2012). To better locate the differences in coupling angular strength of this PES we then expand the RR PES in terms of Legendre Polynomials:

(1) $$V^{RR}(R,\theta )=\sum_{\lambda }^{\lambda_{max}}V_{\lambda }^{RR}(R)P_{\lambda }(\cos(\theta ))$$

where the radial multipolar coefficients are obtained by a well-known numerical quadrature:

(2) $$V_{\lambda }^{RR}(R)=\int_{-1}^{1}V^{RR}(R,\theta )P_{\lambda }(\cos(\theta ))d\cos(\theta )$$

**Figure 3:**
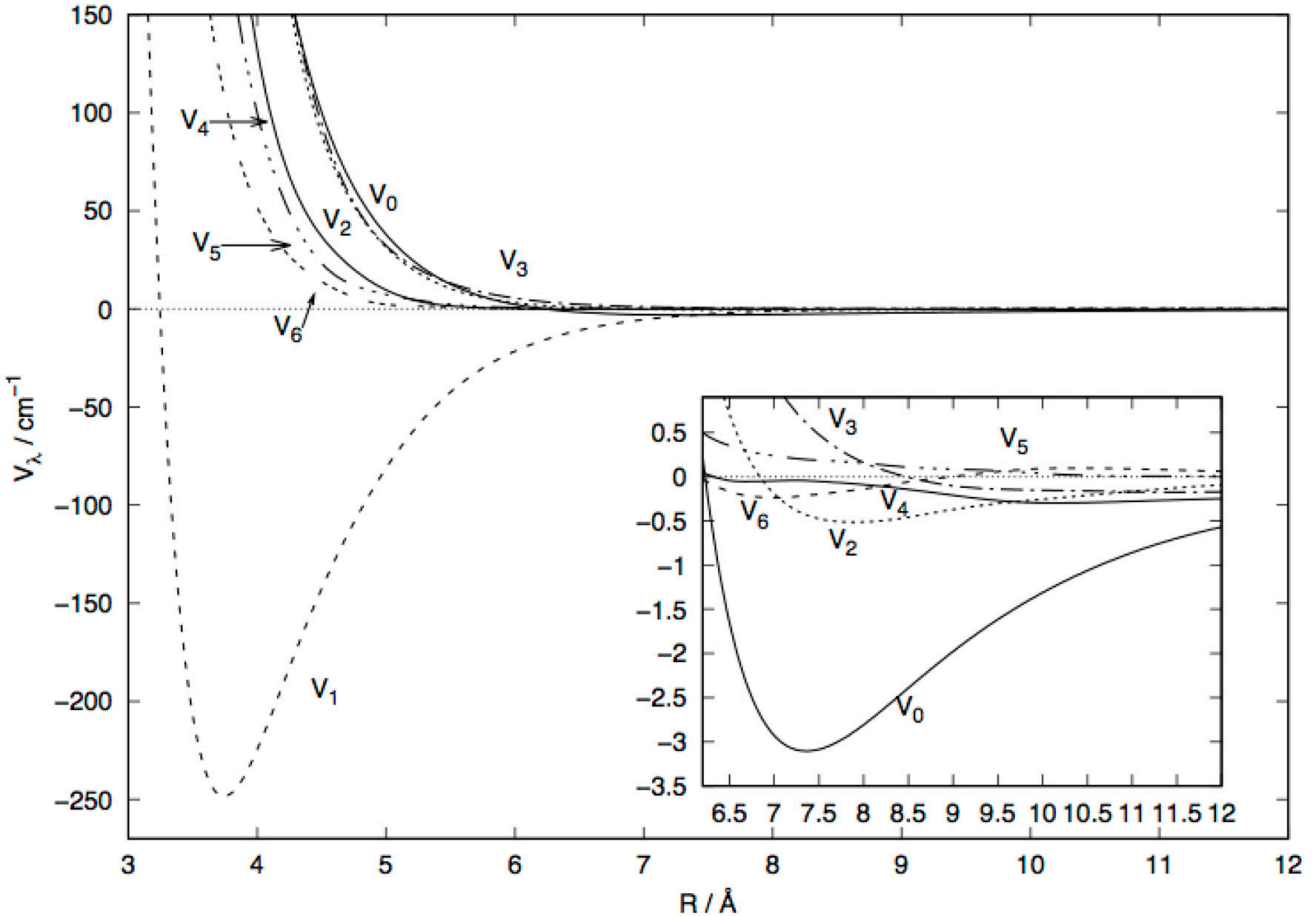
Radial behavior of the lower seven multipolar coefficients for the MgH^−^ (X^1^Σ^+^) plus He discussed in the present work. See main text for further details.

Because of the strong orientational anisotropy of the present system, the index of the sum of equation 1 was required to extend up to λ_*max*_=20 to generate converged coefficients. A pictorial view of the radial features of those coefficients, from λ=0 to λ=6, are reported in Figure 3.

In the main figure one sees that the λ=1 coefficient is the strongest one in the region closer to the molecular anion and it keeps its attractive features down to the shortest distance from the center of mass (c.o.m.) of the molecular anion. The smaller panel shows an enlargement in the region of the larger distances where most of the higher multipoles are repulsive or only weakly attractive. One sees from it that the spherical term, the λ=0 coefficient, is only very weekly attractive, and even more weakly so is the λ=2 coefficient. All other terms are essentially repulsive and show the outset of their repulsive walls at fairly large distances. Thus, we could say that the λ=1 coefficient dominates the short-range interaction while all other coefficients are strongly repulsive and exhibit the starting of their repulsive features at larger distances.

**Figure 4:**
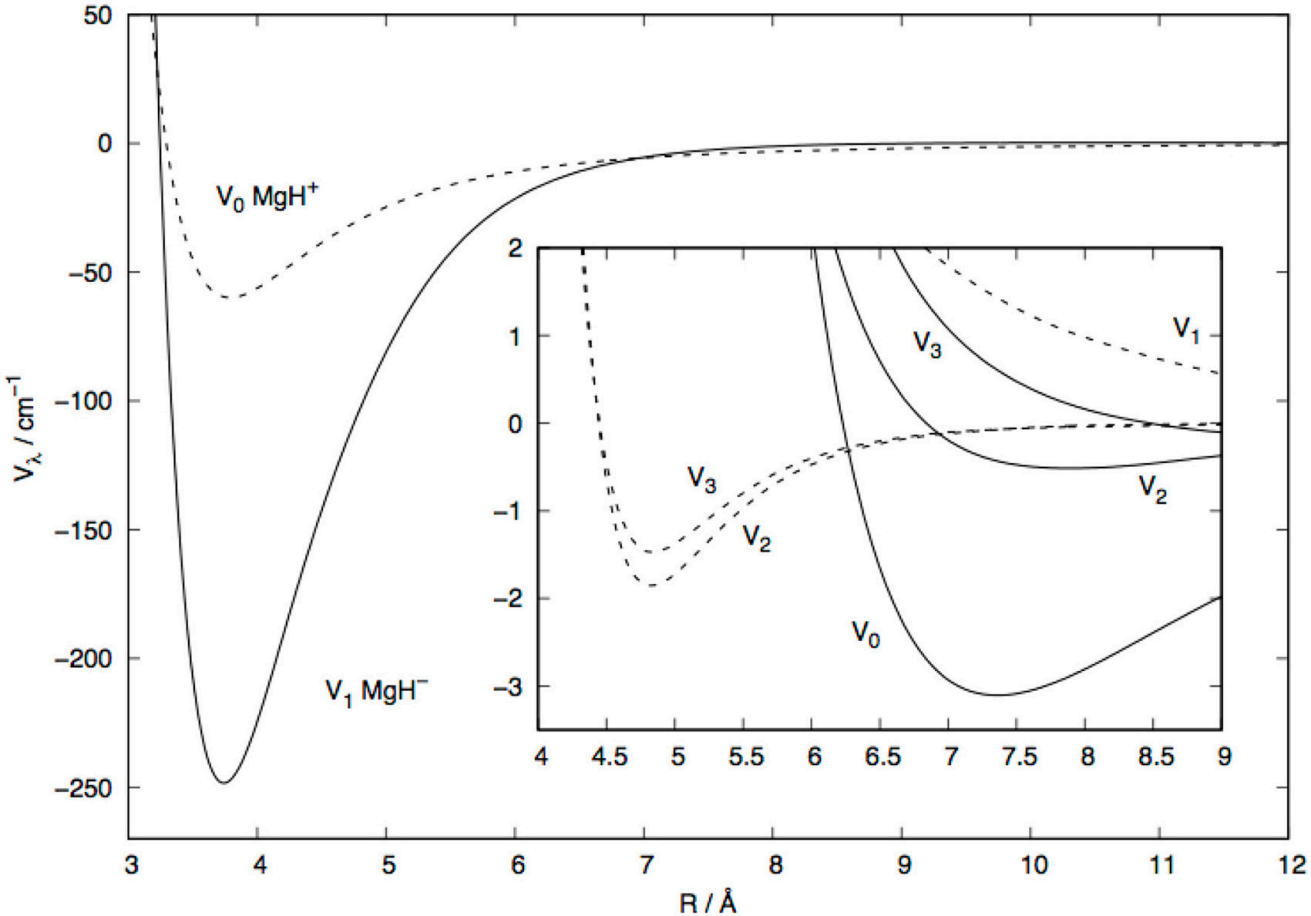
Computed radial multipolar coefficients for MgH^−^/He system (solid curves) and the MgH^+^/He system (dashed curves). The latter data are taken from González-Sánchez et al. (2018a). The inset shows the enlarged view of the coefficients at larger distances.

An interesting comparison with the behavior of the same multipolar coefficients but for the case of the closed-shell cation, the MgH^+^ (X^1^Σ^+^) partner to the He atom, is shown by the data of Figure 4: the anion's multipolar coefficients are given by solid lines, while those for the cation are shown by dashed curves. The following considerations could be had by looking at the data reported in this figure:

1. In the short-range region of relative distances we see that the dominant coefficient for the MgH^−^ target is the one controlled by the *P*_1_(cos(θ)) polynomial, while for MgH^+^ the spherical term for λ=0 is the strongest coefficient;
2. The next higher coefficients, with λ=2 and λ=3, are similar in strength for both systems although those for the anionic target penetrate less closely to the c.o.m. that those for the cations;
3. The anionic coefficients are on the whole stronger in the inner region than those for the cation's, but the latter multipolar coefficients show a more limited region of interaction where the He atom is excluded by repulsive forces, thereby indicating a deeper penetration of the neutral projectile into the interaction region of the cation. The excess negative charge on the anionic molecular partner causes the He atom to experience a larger region of the interaction where repulsive around the molecular target dominate.

**Figure 5:**
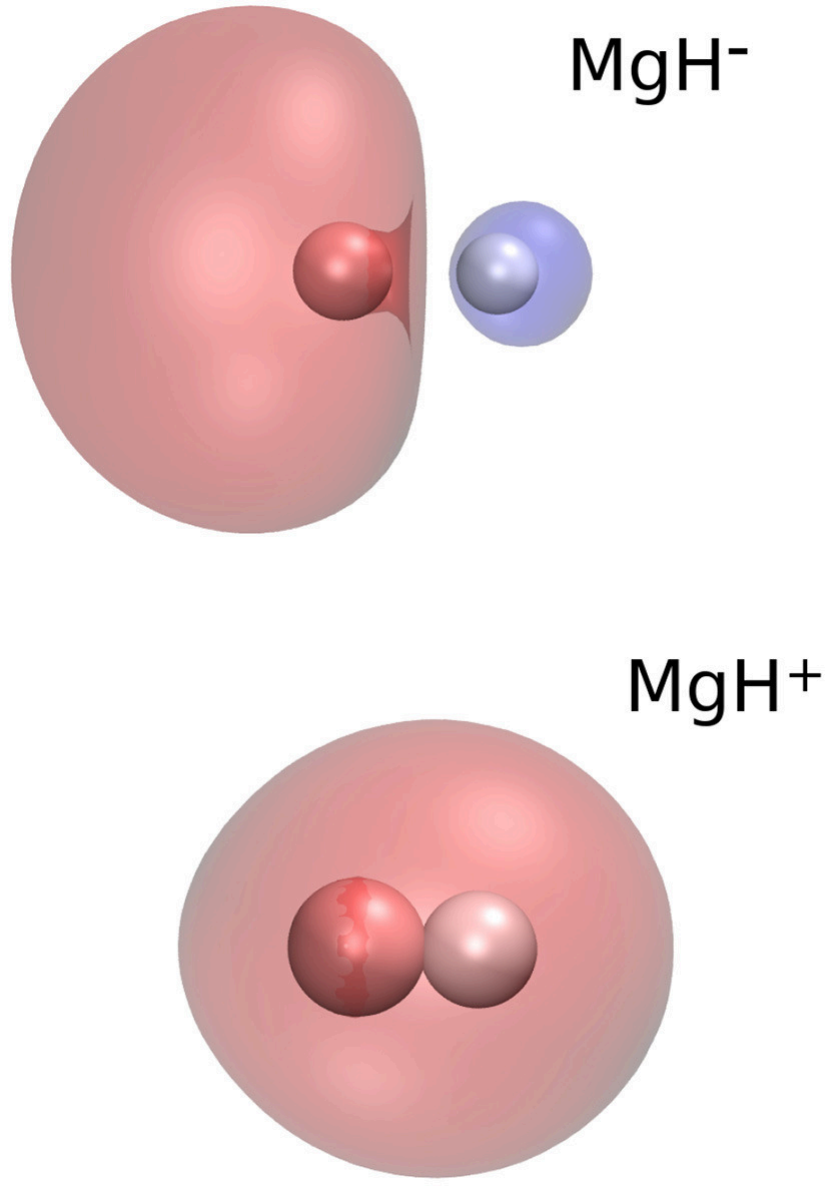
Computed 3D electronic densities for the two, doubly occupied, HOMOs associated with the X^1^Σ^+^ electronic states of MgH^−^ **(Upper)** and of MgH^+^ **(Lower)**. The Mg atom is located on the left of both panels.

A qualitative justification for these differences in the behavior of the two PESs which we are comparing here could be had by looking at the spatial shape of the electron densities of the highest occupied molecular orbitals (HOMOs) pertaining to the two different molecular ions. These are shown as 3D pictures in the two panels of Figure 5.

It is interesting to note that the extra electron in the doubly-occupied MO of MgH^−^ is essentially located along the bond of the molecule and as a σ orbital, for which the *P*_1_(cos(θ)) symmetry is dominant. On the other hand, the most diffused (also doubly occupied) MO for the cation partner is clearly of σ-symmetry and presents a reduced orientational anisotroy of this external charge region to the incoming He atom. Thus, we could surmise that the V_1_(*R*) coefficient for the MgH^−^/He interaction is more important than its V_0_(*R*) contribution, while the opposite occurs for the MgH^+^/He interaction, as shown by Figures 3, 4.

Another interesting piece of information could be gathered from the behavior of the interaction forces in the long-range (LR) region as given by the perturbative expansions of the expression for the intermolecular forces (Stone, 2013):

(3) $$V(R,\theta |r_{eq})\overset{R\to \infty }{=}V_{LR}(R,\theta )\sim -\frac{\alpha_{He}}{2R^{4}}-2\alpha_{He}\frac{\mu P_{1}(\cos\theta )}{R^{5}}$$

(4) $$-\frac{\alpha_{He}\mu^{2}}{R^{6}}\;$$

(5) $$\begin{array}{l} -\left(\alpha_{He}\mu^{2}+Q\alpha_{He}\right)\frac{P_{2}(\cos\theta )}{R^{7}}\;\\ ... \end{array}$$

Where α_*He*_ is the dipole polarizability for the neutral He atom (1.3837 $a_{0}^{3}$; Masili and Starace, 2003) and μ the electric dipole moment of MgH^−^ (X^1^Σ^+^) (0.733 a.u.; González-Sánchez et al., 2017). In the present instance, the positive value of the dipole moment dominates the interaction in the outer radial region where the PES can become attractive. This feature is adding to the attractive features of the V_0_ term which is important in the same radial region where the V_1_(R) becomes repulsive.

On the other hand, the MgH^+^ partner has a larger dipole moment (1.44 a.u.; Sadlej and Urban, 1991) but, directed from the positive centroid of charges to the negative one, hence along the positive direction of the z-axis (Sadlej and Urban, 1991). This implies a negative sign in equation 3, i.e., opposite to the case of MgH^−^. The result, together with the smaller spatial region where the coefficients in equation 1 become repulsive, is that the spherical term dominates the multipolar expansion as shown by Figure 4.

In conclusion, given the differences shown by the present anionic PES with respect to that of its cation, we expect that the collisional dynamics in cold traps involving state-changing processes will be different for the present system. Such differences will be presented and discussed in the following sections.

## 3. The Quantum Dynamics of State-Changing Collisions

The inelastic quantum dynamics we wish to study now involves solely the rotational levels of the anionic target, while it is considered to be in its ground vibrational level after the preparation in the cold traps (Hauser et al., 2015). The rotational constant of the MgH^−^ rigid rotor (RR) is 5.6988 cm^−1^ (González-Sánchez et al., 2017), which is slightly smaller than the one for the corresponding cation MgH^+^: 6.3870 cm^−1^ (Sadlej and Urban, 1991).

**Figure 6:**
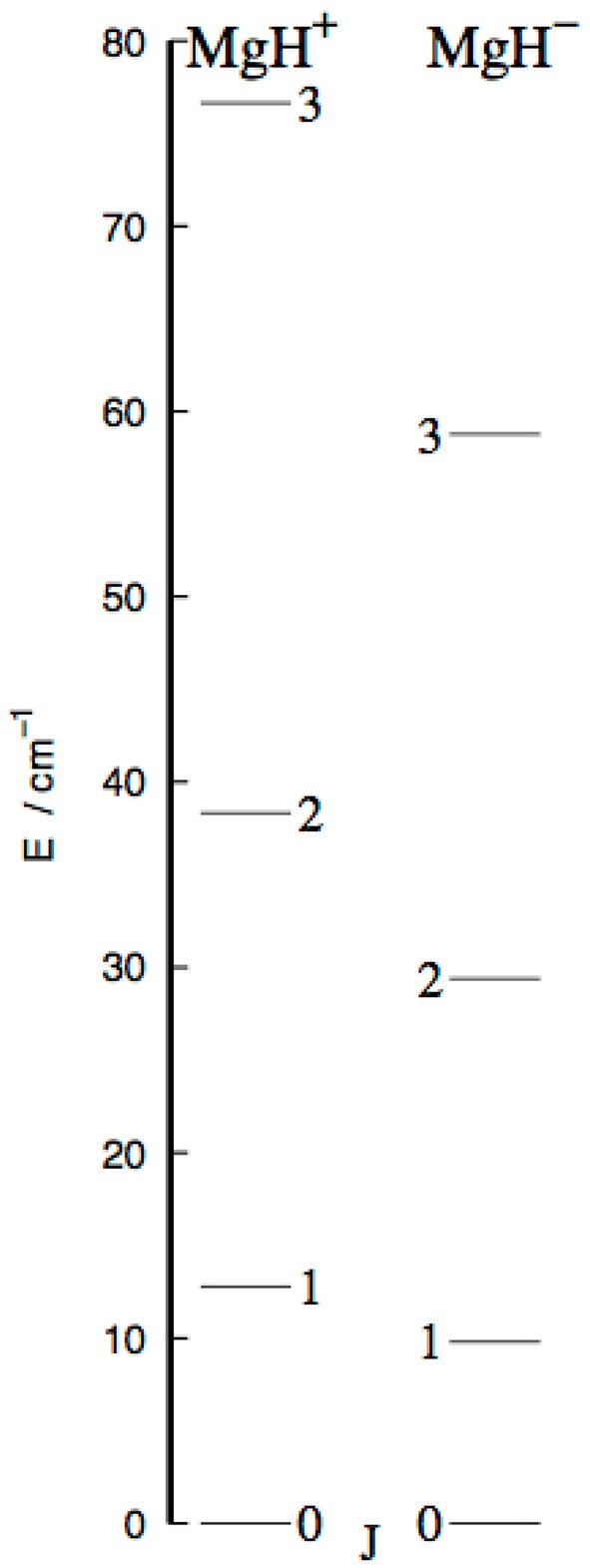
Computed energy spacings between the lowest four rotational levels for the MgH^+^ **(Left)** and MgH^−^ **(Right)** molecular ions. See main text for further details.

In Figure 6, we report a pictorial view of the involved energy levels for the X^1^Σ^+^ electronic states of the two molecular ions.

It is interesting to note that the present anion shows smaller energy gaps between its lower levels, those which we expect to be involved in possible experiments of selective photo-detachment (Hauser et al., 2015).

This property is likely to play a role when the relative rates for state-changing dynamics will be discussed below in comparison with the MgH^+^/He system which we have recently analyzed (González-Sánchez et al., 2018a).

The next computational step involves solving the quantum inelastic dynamics of MgH^−^ collisions with He atoms using the time-independent formulation of the multichannel coupled scattering eq.s. This method involves the well-known coupled-channel approach subject to the standard boundary conditions, the one leading to the calculation of the matrix elements of the full scattering S-matrix (Taylor, 2012). For this purpose, we have employed our in-house numerical code ASPIN and details of its implementation have been given before (López-Durán et al., 2008; González-Sánchez et al., 2015). We therefore do not discuss it again in the present work. Suffice it to say that the physical observables which we obtain from the ASPIN scattering code are in this case the state-to-state partial cross sections for each of the contributing total angular momentum *J*: $\sigma^{J}\left(j^{\prime }\leftarrow j|E_{i}\right)$, with *E*_*i*_ giving the initial relative energy between partners. The further summation over the contributing angular momenta (which, in the present case, was take up to *J*_*max*_ = 50) will therefore yield the corresponding state-to-state partial integral cross sections:

(6) $$\sigma (j^{\prime }\leftarrow j|E_{i})=\sum_{J}^{J_{max}}\sigma^{J}(j^{\prime }\leftarrow j|E_{i})$$

From them we can further obtain the partial rotational quenching and heating rate constants, $K_{jj^{\prime }}\left(T\right)$ at the temperature of interest:

(7) $$K_{jj^{\prime }}(T)=\int \sigma (j^{\prime }\leftarrow j|E)\sqrt{\frac{4E}{\pi {\left(k_{B}T\right)}^{3}}}\exp(-E/k_{B}T)EdE$$

We have integrated the computed cross sections over an extended range of collision energies for the corresponding cross sections, ensuring that the threshold behavior is well-described by a dense grid of values. We have further used and extended the range of energies well beyond that necessary to map the required interval of temperatures. Numerical convergence has been checked to have reached more than 0.01 stability of the final rates.

The results from the above calculations will be presented and discussed in the following section.

## 4. Rotationally Inelastic Processes and Kinetic Evolution in the Trap

### 4.1. Behavior of State-Changing Cross Sections

**Figure 7:**
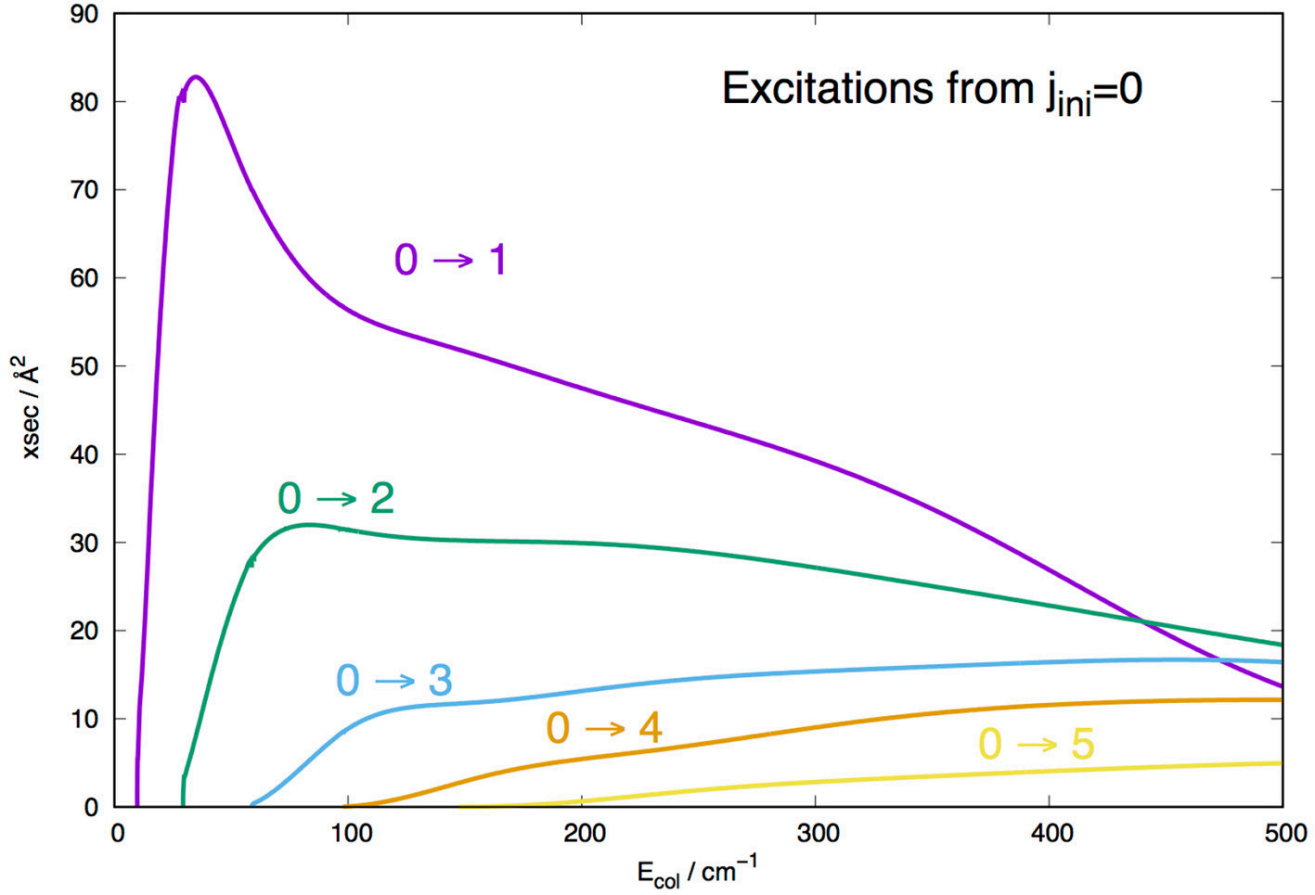
Computed rotational excitation cross sections for MgH^−^ (X^1^Σ^+^) in its *j*=0 initial rotational level. See main text for further details.

**Figure 8:**
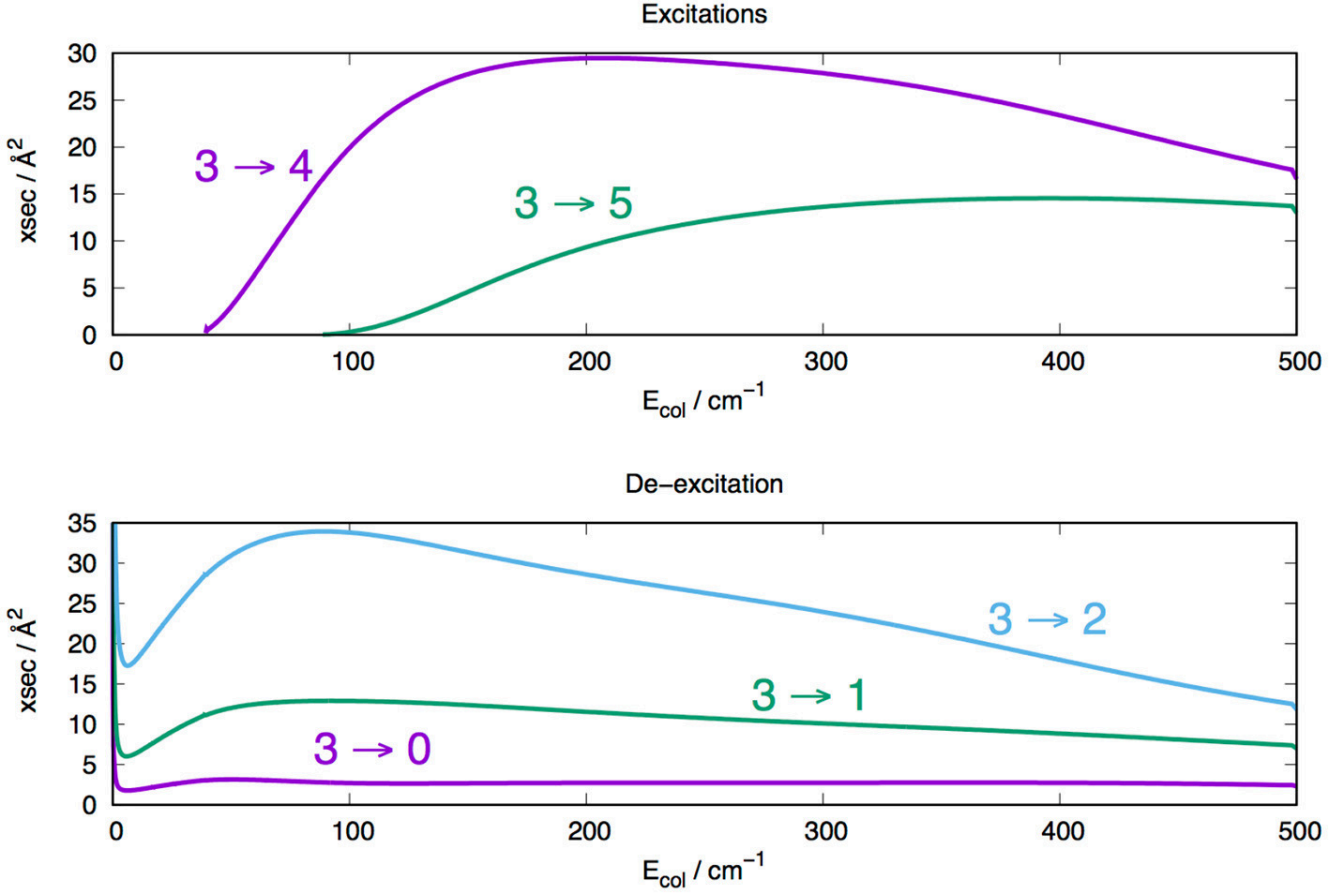
Computed state-changing rotationally inelastic cross sections for the MgH^−^ target in its *j*=3 initial. **Upper**: excitation cross sections. **Lower**: de-excitation cross section.

The radial integration for the cross sections of Equation (6) was extended out to *R*_*max*_ = 1000 Å, while the angular anisotropy of the interaction potential of Equation (1) was extended out to λ_*max*_ = 19 in order to guarantee numerical convergence of the state-to-state inelastic probabilities included in the present treatment. We actually computed a broad range of elastic and inelastic cross sections which were employed to generate the corresponding rates from Equation (7). However, we present in Figures 7, 8 a small sampling of them to show briefly their general sizes and energy dependence for excitation and de-excitation processes.

The data reported in the Figure 7 show clearly the strong dominance of the Δ*j*=1 cross sections with respect to all the other rotational excitation processes. The λ=1 coupling term of the PES shown by Figure 3 is the strongest coupling term between target levels, a feature which directly reflects on the (0 → 1) excitation cross section being the largest at nearly all energies considered in that Figure 7. In the region of interest, i.e., around 100–200 cm^−1^, we see that the increasing of the energy gaps between levels involved in Δ*j* = 1 transitions causes the corresponding cross sections to become uniformly smaller: all the direct coupling terms with λ > 1 are seen in Figure 3 to be of similar strength and all chiefly acting in the outer radial region, so that the changes in the energy gap chiefly control the relative sizes of the corresponding inelastic cross sections.

The data reported by Figure 8 involve both the excitation cross sections (upper panel) and de-excitation cross sections (lower panel) for the case of the molecular target being initially in its *j* = 3 rotational state. The dominance of the Δ*j* = ±1 propensity rule for the size of the cross sections is clearly visible in both panels, while the increasing of the energy gaps between levels is again causing the size of the cross section to decrease as the Δ*j* values increase.

**Figure 9:**
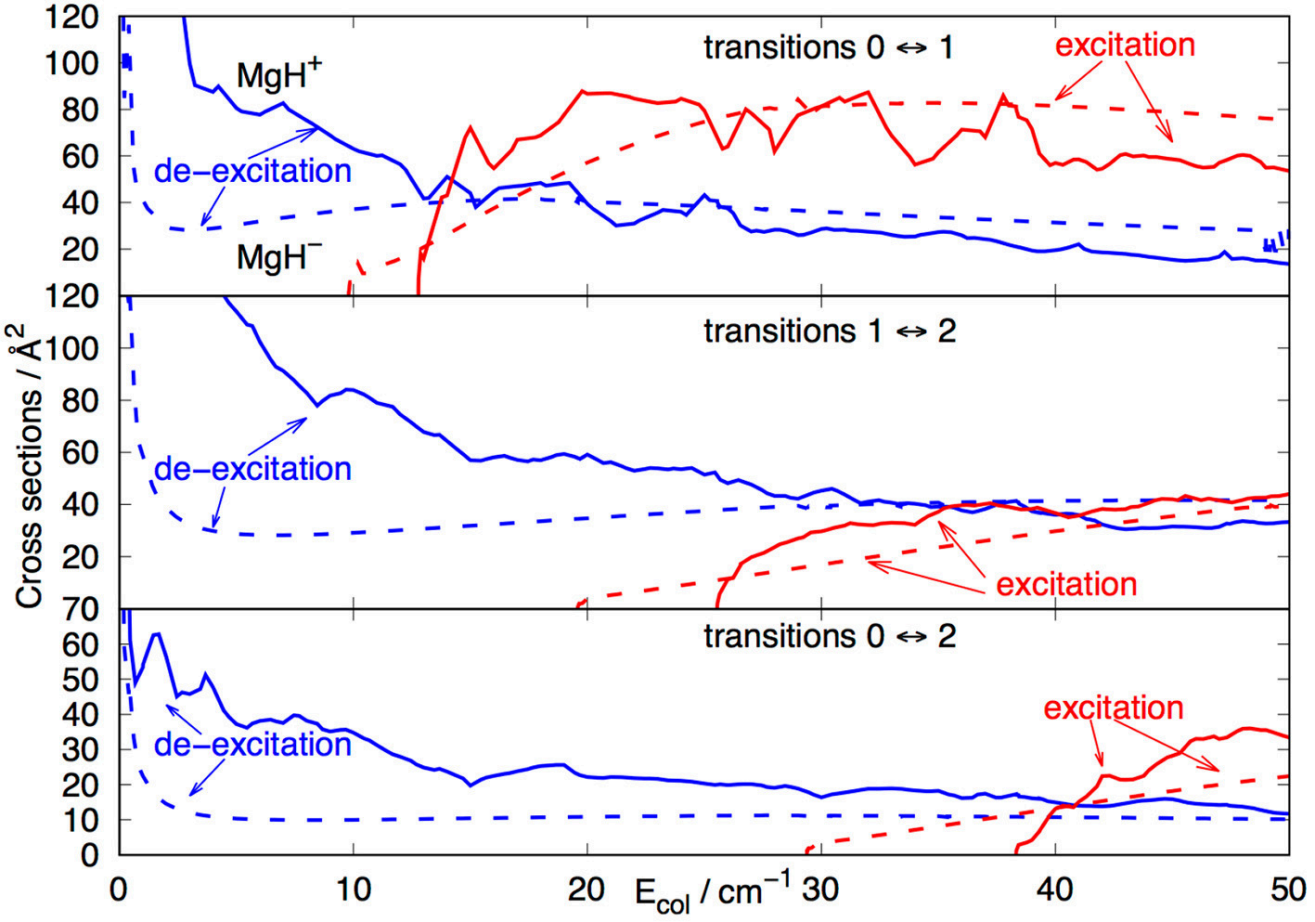
Comparison between state-changing cross sections for rotational transitions in MgH^+^ (solid lines) and in MgH^−^ (dashed) in collision with He atoms. See main text for further details.

At least for the case of the three lowest rotational levels of the target molecule, it is also interesting to make a comparison between the cross sections behavior in the case of MgH^−^ in the trap (present results) and those obtained for the case where the cation is instead in the trap (discussed by us in more detail in González-Sánchez et al., 2018b). The comparison is reported by the three panels of Figure 9 below.

The top panel in that figure shows both the excitation and de-excitation cross sections involving the lowest two rotational levels of MgH^+^ (solid lines) and MgH^−^ (dashed lines). We have seen before (see Figure 6) that the energy gap is smaller for the anion in comparison with the cation. On the other hand, the direct coupling potential, the V_1_(R) multipolar coefficient in Figure 3, is stronger for the anion than for the cation case. The present results however indicate that the excitation process produces fairly similar cross sections for the two systems, although the cation's cross sections are larger at threshold energies. Since we are dealing with low collision energies, the range of action of the PES also plays a significant role. From the data in Figures 2, 3, we see that the He atom gets much closer to the MgH^+^ target than it does for the MgH^−^ case, thereby reducing the radial extension of its torque effects in the latter case with respect to the former. Furthermore, the LR forces of Equations (3–5) have opposite signs of their dipolar coefficients in Equation (3), with the MgH^+^ being overall positive with respect to MgH^−^ which remains negative. This would imply slightly weaker LR contributions for the cation in relation to the anion. Such different features contribute to the threshold differences in size and behavior of the cross sections between these two molecular ions.

The data in the middle panel report now the excitations and de-excitation cross sections involving the Δ*j*=±1 transitions between |*j* >= 1 and |*j* >= 2 levels. The relative behavior remains substantially the same, and for the same reasons listed above, with the cation exhibiting markedly larger cross sections for de-excitation processes near threshold.

Finally, the data in the bottom pane involve the Δ*j*=±2 transitions between the lowest possible levels: |*j* >= 0 and |*j* >= 2. Here again the cross sections are fairly similar in size, with the exception of the near-threshold de-excitation cross sections which are again larger for the MgH^+^ target in comparison with the MgH^−^ case.

In conclusion, we found that both ions behave fairly similarly, in spite of their structural differences, when looking at state-changing cross sections. The main difference being the larger de-excitation cross sections for the cation at energies very near the energy threshold. Such differences, however, can play a definite role when comparing state-changing rates at the low temperatures of an ion trap. These effects will be further discussed in the following subsection.

### 4.2. Computed Inelastic Collisional Rates

By a numerical quadrature of the computed cross sections of equation 6, as indicate by equation 7, we can obtain the corresponding rates as a function of trap's temperature (González-Sánchez et al., 2015, 2018b; Schiller et al., 2017).

**Figure 10:**
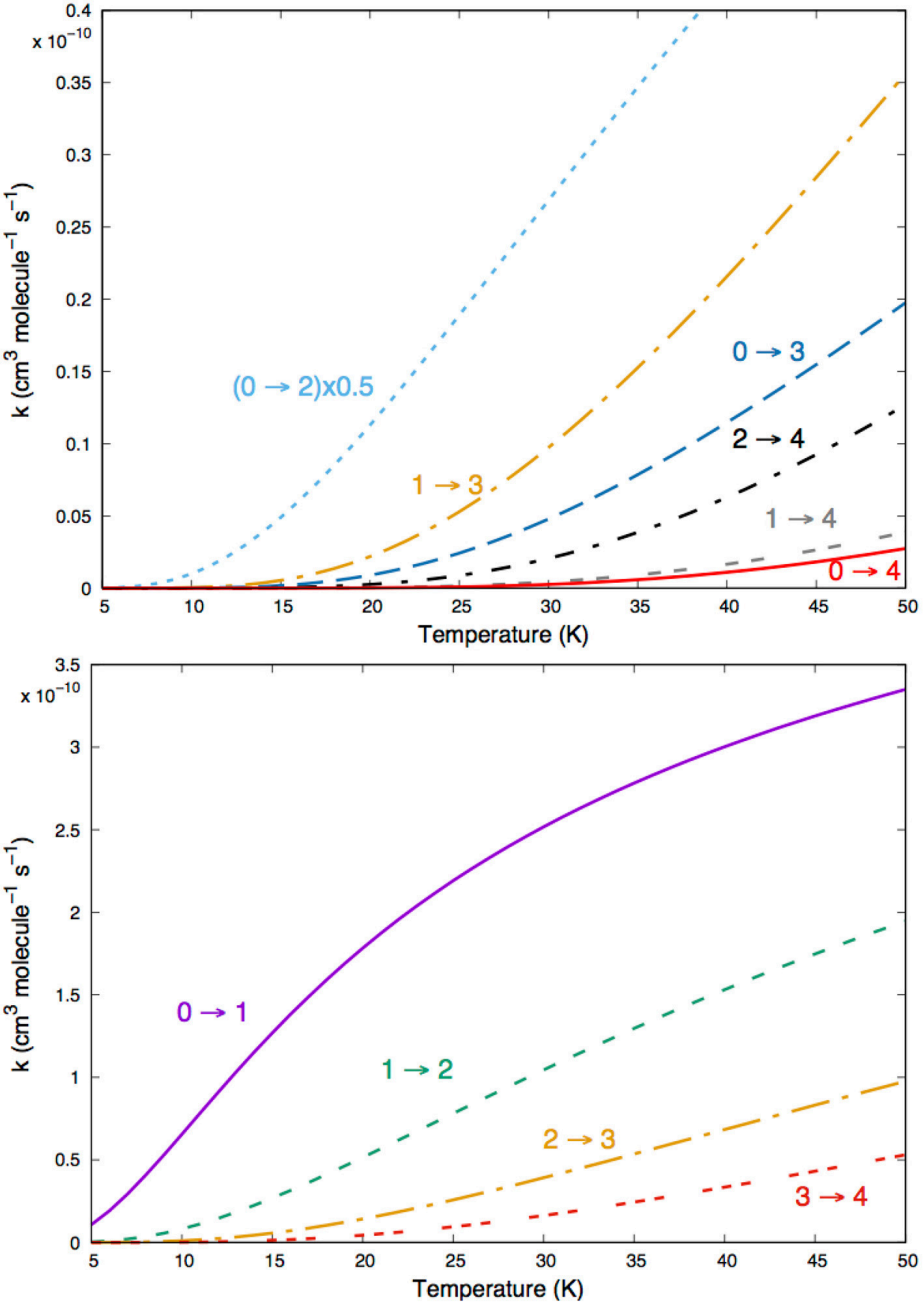
Computed state-changing excitation rates among the lowest five rotational levels of MgH^−^ (X^1^Σ^+^) in collision with He atoms.

**Figure 11:**
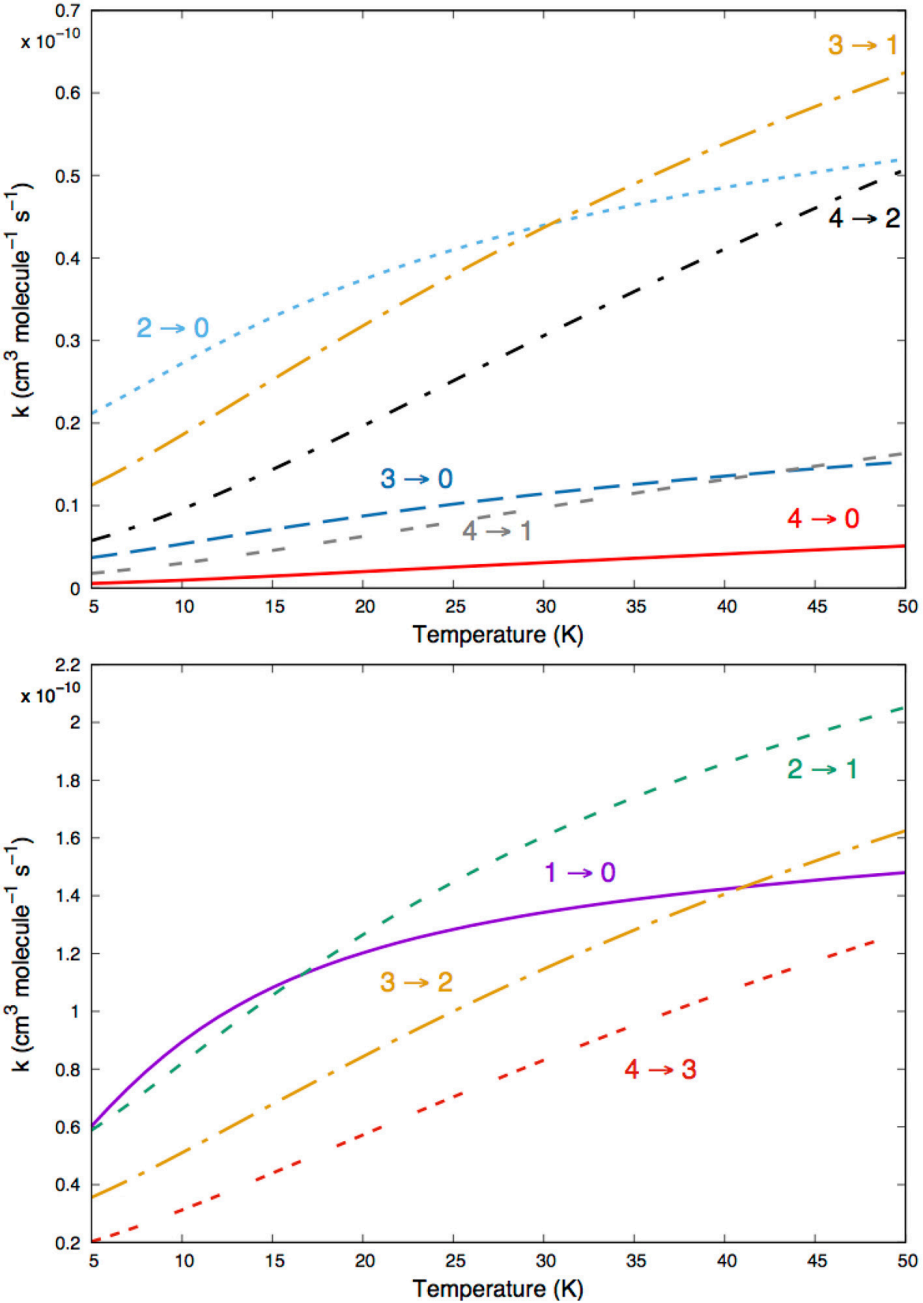
Computed de-excitation rotationally inelastic rates for the same states of Figure 10.

The data shown by Figure 10 report the computed rates for excitation processes between the lowest five rotational states, of MgH^−^ (X^1^Σ^+^) that we expect to be involved in the cold ion traps of the planned experiments (Hauser et al., 2015; Gianturco et al., 2018). Those given by Figure 11 describe the corresponding de-excitation transition rates between the set of rotational states of the rotationally cooling anion in the trap. The following considerations can be made by a perusal of the figures data:

1. the single-jump excitations (Δ*j*=1) in the lower panel of Figure 10 are all at least one order of magnitude larger than the Δ*j*=2 transitions on the upper panel. Clearly the dominant strength of the coupling potential coefficient with λ=1 (see Figure 3 controls the efficiency of the direct collisional torque applied to the molecular rotor during the low-T collisions;
2. The increasing of the energy gaps during the excitation processes (see upper panel in Figure 10) causes the corresponding inelastic rates to become smaller: the (0 → 4) state-changing rate at 30 K, for example, is nearly two orders of magnitude smaller than the inelastic rate for the (0 → 1) process in the lower panel of Figure 10;
3. Very similar types of behavior could be seen for the state-changing rotation-cooling (de-excitation) rates shown by the data in the two panels of Figure 11: the Δ*j*=-1 transitions produce by far the largest rates over the selected range of temperatures. On the other hand, one also sees there that all rates are in the range of 10^−10^ cm^3^ s^−1^, i.e., similar in size to those found earlier for the MgH^+^ cation in cold traps under similar conditions (González-Sánchez et al., 2018b).

**Figure 12:**
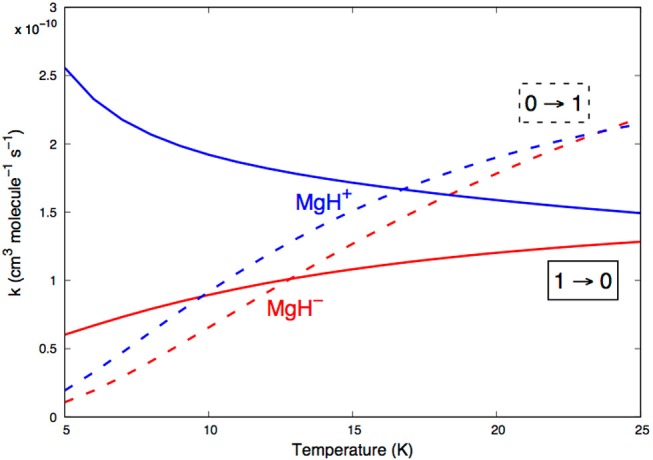
A comparison between computed state-changing inelastic rates involving the lowest two levels of MgH^−^ and MgH^+^. See main text for further details.

To further investigate the comparative behavior of the two systems, we present in Figure 12 a direct comparison between excitation and de-excitation rates involving transitions between the two lowest rotational states of MgH^−^ and MgH^+^ molecular ions.

By looking at the two excitation rates shown in the figure, we see that the differences in structural features between the two PESs have little influence of the final rates: the (0 → 1) excitation rates are of the same order of magnitude, with that pertaining to the MgH^−^ partner remaining invariably somewhat smaller in the lowest range of temperatures. As discussed earlier, this system shows a reduced radial range within which the Δ*j*=1 torque can act in comparison with that acting for MgH^+^. As a consequence, we see the smaller values for the corresponding rates.

This difference is even more marked when we look at the rotation-cooling (de-excitation) rates between the lowest two rotational states. The rates for the MgH^−^ partner are around 50% smaller than those shown by the MgH^+^ cation, especially over the lowest range of temperatures up to about 15 K.

### 4.3. Collisional Evolution of Rotational Population

In order to gain more insight into the kinetic effects of population-changes in the trapped anions after the uploading of the He atoms, and as it was recently demonstrated by work in our group (Hauser et al., 2015; Hernández Vera et al., 2018), it is possible to manipulate experimentally the relative populations of the rotational quantum states of a rigid rotor confined in a cold ion trap. The method, which can also be applied to the present problem, consists in depleting by an intense photo-detachment laser one or more of the lower excited rotational states of the anion while however leaving the population of its ground state intact. During the corresponding computational modeling of the physics involved one usually neglects any induced or spontaneous radiative dipole transition in the molecular anion. This is justified by noting that, given the low temperature of the black-body radiation distribution in the trap, and the usual smallness of the Einstein coefficients found for the radiative emissions between the lower rotational levels in earlier work (Schiller et al., 2017), we do not expect that such radiative processes would play here a significant role in competition with the collisional depletion paths.

The initial step is therefore that of writing down the master equations which follow the rotational state population changes by collisional energy transfers:

(8) $$\frac{dn_{i}(t)}{dt}=\sum_{j\neq i}n_{j}(t)C_{ji}-n_{i}(t)\sum_{j\neq i}P_{ij}$$

where *P*_*ij*_ is the destruction rate coefficient for level *i*, with its formation rate given by the *C*_*ji*_ coefficient. They are given by:

(9) $$P_{ij}=n_{He}K_{i\to j}(T)+K_{PD}$$

(10) $$C_{ji}=n_{He}K_{j\to i}(T)$$

Equation (9) contains both the collisional state-changing rate *K*_*i*→*j*_(*T*) and the $K_{PD}\left(s^{-1}\right)$ photo-detachment rate for the situation where the molecular ion population is further altered by switching on a photo-detaching laser source after reaching an equilibrium distribution by pure collisional evolution of the rotational state populations. In the present modeling we will focus on the collisional evolution of the system and initially take the *K*_*PD*_ rate to be equal to zero (no laser has been switched on). The steady-state solution of the kinetic eq.s is reached when the populations *n*_*i*_(*t*) get to their values when *t* ~ ∞. They are found by solving equation 8 upon setting *d***n**(*t*)/*dt* = 0 and therefore solving the resulting algebraic equations. We also verified that the steady-state population fractions, for each anionic rotational level, correspond to a Boltzmann distribution with *T*_*rot*_ = *T* (Schiller et al., 2017).

**Figure 13:**
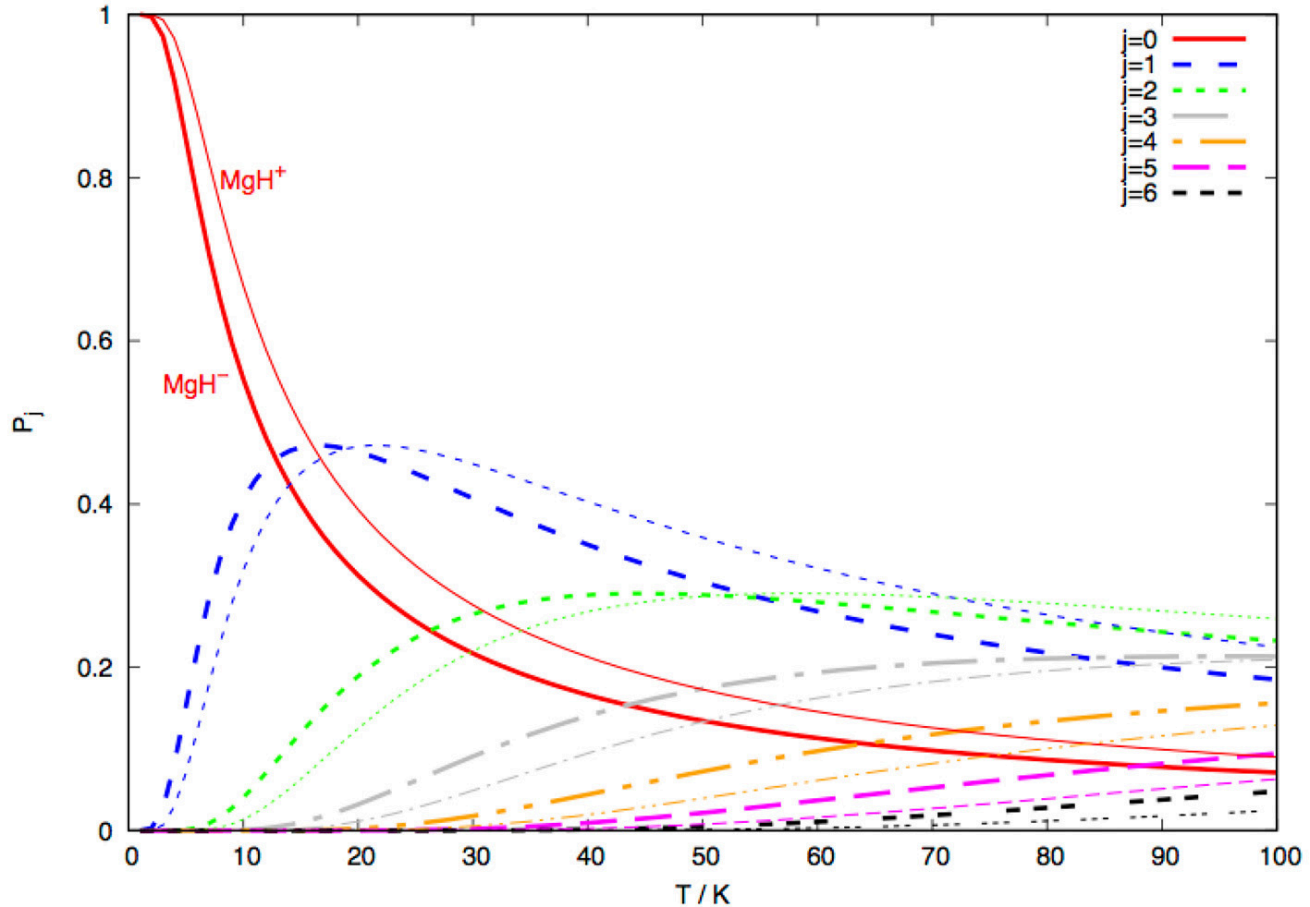
Computed steady-state fractional populations of the rotational levels from *j* = 0 to *j* = 6 for the MgH^−^ anion (thick lines) in comparison with the fractional populations for the same set of levels for the MgH^+^ (thin lines). See main text for further details.

The data reported by Figure 13 show the steady-state populations for the lowest seven rotational levels of MgH^−^ (X^1^Σ^+^) over a range of temperature up to 100 K.

In that same figure we also show the steady-state population which has been achieved in our calculations for the corresponding MgH^+^ (X^1^Σ^+^) cation under the same dynamical conditions (González-Sánchez et al., 2018a). We see clearly that, for temperatures up to about 15 K, the fractional population of the |*j* >= 0 state in MgH^−^ is about 40%, while that of the |*j* >= 1 state is about 45% and the |*j* >= 2 state is around 15%. It is around T = 2–4 K that all anions are occupying the |*j* >= 0 level by more than 98%. The cationic molecule, on the other hand, has reached at 15 K a fractional population for the |*j* >= 0 level around 50% and around 43% for its |*j* >= 1 level. On the whole, in fact, we see that the collisional state-changing of rotational level fractional populations, after He atom uploading in the trap, is more efficiently occurring with MgH^+^ than for MgH^−^, although both systems show similar steady-state behavior of their fractional populations.

**Figure 14:**
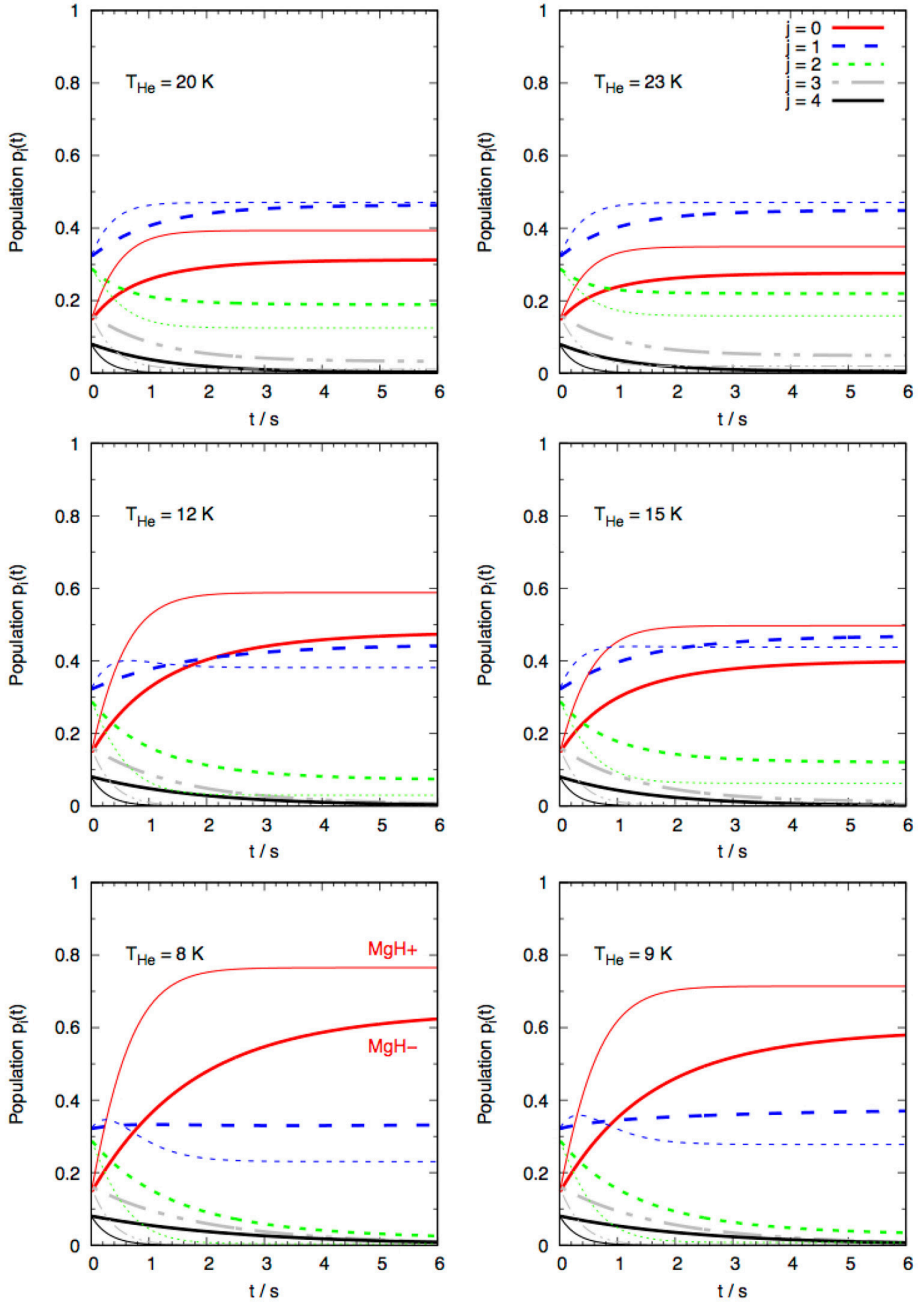
Computed time evolutions of the fractional rotational state populations for MgH^−^ (thick lines) and MgH^+^ (thin lines) ionic molecules in cold traps after uploading of the He buffer gas. Six different *T* values are considered and the lowest five levels examined.

Another way of further comparing the collisional evolution of rotational level populations in the cold trap (the purely collision-driven evolution) is to compute the changes of population fractions at fixed T values and as a function of time after the buffer gas upload. The results are shown by the six panels reported by Figure 14.

The data of Figure 14 examine six different values of *T*, following those considered by the earlier experiments on the MgH^+^/He system (Hansen et al., 2014). The following comments can be made by considering the data in the six panels, where only the lowest five rotational state fractional populations are shown:

1. At the lowest temperatures considered in this study (lowest two panels in figure) we see that the MgH^+^ cation reached steady-state conditions after about 2 *s*, while the MgH^−^ species appear to take much larger for the |*j* >= 0, 1, and 2 states to reach stationarity;
2. At these low temperatures we also see that the fractional population of the |*j* >= 0 ground state is much higher for MgH^+^ than it is for MgH^−^, for which the two lowest levels turn out to have more comparable fractional populations;
3. When the higher temperatures are considered, we see that only around 20 K and above the MgH^−^ fractional populations achieve steady-state conditions within 3–4 *s*. We also see that the fractional population of its |*j* >= 0 state remains smaller than that reached by the MgH^+^ system, while the next higher rotational state with |*j* >= 1 reaches for both ionic molecules a steady-state population close to 50%.

On the whole, therefore, we can say from the above numerical experiments that the MgH^−^ anion would be less efficient than it cationic counterpart in achieving steady-state conditions in the trap and that lower temperatures would be needed to attain 80% fractional population for its rotational ground state.

## 5. Summary and Conclusions

In the present work we have analyzed in some detail the quantum dynamical modeling of the fractional rotational population of a molecular anion, the MgH^−^(X^1^Σ^+^) which can evolve within a cold ion trap environment after the collisional interaction that follows the uploading of He atoms as a buffer gas. The population density of the uploaded gas has been taken to be of 10^10^ cm^−3^ as discussed by the experiments involving its corresponding cation MgH^+^(X^1^Σ^+^) (Hansen et al., 2014). Our calculations involved the evaluation from first principles of the PES of the electronic energy for the molecular anion and the He atom in the trap. Thus, we were able to analyze in detail the orientational anisotropy of that potential and could then compare it with the features of the same type of interaction between the He atom and the molecular cation MgH^+^ which we had computed and analyzed in our earlier work (González-Sánchez et al., 2018a). The major differences between the two PES indicate more repulsive effects coming from the extra electron of the molecular anion, which then cause the presence of a sort of “excluded volume” around the molecule for the approaching He atom which is larger for the anionic partner than it is for the cationic one. The different directional features of the permanent dipoles are also found to affect the relative strength of the orientational forces which dynamically drive the collisional state-changing processes.

The quantum, time-independent multi-channel approach is employed to treat the coupled-channel (CC) scattering problem and up to six different rotational states of the target are coupled during the dynamical study of the partial, state-to-state inelastic cross sections at the relevant collision energies.

The comparison with the same set of data for the MgH^+^/He set up in the cold trap indicates that, although their inelastic cross sections are fairly similar in relative sizes and in energy dependence, the MgH^+^ turns out to be more efficiently cooled by collisions to its lowest rotational state than the anionic counterpart. As a result of these differences, we found that the dominating state-changing rates are in both case those for the Δ = ±1 processes and that down to T values < 30 K the anionic molecules show smaller inelastic rates than those found for the cation.

We have further studied the time evolution of the fractional populations of the lower rotational states in order to model the collisional preparation of the anionic molecule in the trap to further perform state-selected photo-detachment experiments as already done in our group for OH^−^/He (Hauser et al., 2015) and for ${\text{NH}}_{2}^{-}$/He (Gianturco et al., 2018; Hernández Vera et al., 2018; Lakhmanskaya et al., 2018). The time evolution of the fractional populations at different temperatures for the trap is shown by our present calculations to be less efficient than in the case of MgH^+^/He and that lower temperatures would be needed to reach a steady-state population dominated by the lowest two rotational states. However, the fact that the collisional state-changing rates for the MgH^−^/He system are still found to be fairly large indicates that such system can indeed be efficiently prepared for state-selective photo-detachment experiments in cold traps. Our next task will therefore be that of modeling the optimal conditions under which laser photo-detachment experiments should be carried out for the present anionic molecule.

## Author Contributions

SG-C and AS were involved in producing the *ab initio* PES used for the quantum dynamics. LG-S carried out the quantum CC calculations. FG suggested the problem and wrote up the initial draft of the presentation of the results. RW with all other authors discussed the physical meaning of the computational findings and contributed to the production of the final version of this paper.

## Acknowledgments

LG-S acknowledges financial support from MINECO (Spain), Grant CTQ2015-65033-P. The computational results were obtained using in-house computer codes running on the HPC infrastructure LEO of the University of Innsbruck. This work was supported by various STSM travelling Grants from COST Action CM1401, held by LG-S. FG and RW thank the support by the Austrian Science Fund (FWF), Project No. 29558-N36.

### Conflict of Interest Statement

The authors declare that the research was conducted in the absence of any commercial or financial relationships that could be construed as a potential conflict of interest.
